# Onscreen presence of instructors in video lectures affects learners’ neural synchrony and visual attention during multimedia learning

**DOI:** 10.1073/pnas.2309054121

**Published:** 2024-03-11

**Authors:** Chanyuan Gu, Yingying Peng, Samuel A. Nastase, Richard E. Mayer, Ping Li

**Affiliations:** ^a^Department of Chinese and Bilingual Studies, Faculty of Humanities, The Hong Kong Polytechnic University, Hong Kong SAR, China; ^b^Princeton Neuroscience Institute, Princeton University, Princeton, NJ 08540; ^c^Department of Psychological and Brain Sciences, University of California, Santa Barbara, CA 93106; ^d^Centre for Immersive Learning and Metaverse in Education, The Hong Kong Polytechnic University, Hong Kong SAR, China

**Keywords:** multimedia learning, socio-emotional processing, neural synchrony, visual attention, individual difference

## Abstract

COVID-19 forced students to rely on online learning using multimedia tools, and multimedia learning continues to impact education beyond the pandemic. In this study, we combined behavioral, eye-tracking, and neuroimaging paradigms to identify multimedia learning processes and outcomes. College students viewed four video lectures including slides with either an onscreen human instructor, an animated instructor, or no onscreen instructor. Brain activity was recorded via fMRI, visual attention was recorded via eye-tracking, and learning outcome was assessed via post-tests. Onscreen presence of instructor, compared with no instructor presence, resulted in superior post-test performance, less visual attention on the slide, more synchronized eye movements during learning, and higher neural synchronization in cortical networks associated with socio-emotional processing and working memory. Individual variation in cognitive and socio-emotional abilities and intersubject neural synchronization revealed different levels of cognitive and socio-emotional processing in different learning conditions. The instructor-present condition evoked increased synchronization, likely reflecting extra processing demands in attentional control, working memory engagement, and socio-emotional processing. Although human instructors and animated instructors led to comparable learning outcomes, the effects were due to the dynamic interplay of information processing vs. attentional distraction. These findings reflect a benefit–cost trade-off where multimedia learning outcome is enhanced only when the cognitive benefits motivated by the social presence of onscreen instructor outweigh the cognitive costs brought about by concurrent attentional distraction unrelated to learning.

## Significance

COVID-19 forced students to rely on online learning. Although the pandemic has subsided, online learning through multimedia instructional videos continues to shape education. This study evaluates the key principles associated with students’ processing of socio-emotional cues in multimedia learning. Our findings support the learning benefits of the social presence of both human and virtual instructors in online videos, with benefits varying across individuals. The data suggest a trade-off mechanism where socio-emotional processing must outweigh concurrent visual distractions to improve learning outcomes. Our findings hold significant implications for today’s education in a digital era, where online video learning is prevalent.

COVID-19 has negatively impacted education in fundamental ways, as students and teachers would lose significant socio-emotional cues when relying exclusively on online learning, as compared with face-to-face classroom learning. However, given its convenience and accessibility, online learning with multimedia instructional videos remains the most popular alternative to in-person classroom education beyond the pandemic, with unprecedented coverage of topics in almost every field. In-depth research in this domain can help better understand the technological features and learner-specific characteristics that contribute to effective video learning (1, 2). In particular, given the prevalence of instructional videos in today’s education, neurocognitive research is needed for evidence-based instructional design grounded in theories of multimedia learning.

According to the multimedia learning framework (3) which advocates the joint use of words and pictures in video learning, more dynamic, interactive, and personable educational videos could yield sustained attention and improved motivation in students. An important hypothesis in multimedia learning is social agency theory (3, 4): Social cues from the instructor and instructional messages can activate the students’ social-affective responses and evoke deeper cognitive processing for selecting, organizing, and integrating information, thereby increasing the effectiveness of teaching and quality of learning. Deep cognitive processing can be realized as the effective use of working memory in processing information. Socio-emotional cues can help to alleviate extraneous cognitive load due to the processing of distracting information (5, 6), thereby promoting deeper cognitive processing.

However, current online learning practices, especially coming out of COVID -19, significantly limit the availability of social interaction and the processing of socio-emotional cues where the instructor typically sits or stands still next to lecture slides. This violates specifically the “embodiment principle” of multimedia learning (3, 7), according to which human faces, facial expressions, and voices are critical for students’ socio-emotional responses and sustained attention. Further, the teacher sometimes turns off the face image and presents only the slides during video lectures. This leads to the consideration of the “image principle” (8–10)—whether the instructor’s onscreen image activates social-affective responses, engages more in-depth cognitive processing, and consequently improves learning. While previous studies have shown positive impacts of embodiment, studies of the image principle, however, are inconclusive, with some showing positive results (8–10) while others suggesting the instructor’s image may distract students, diverting their attention from learning and hence impeding learning (11, 12). The current study is designed to use multimodal experimental paradigms to examine the image and embodiment principles—that is, to determine the effects and educational impacts of the instructor’s onscreen role (i.e., the presence vs. absence of the instructor) and the embodiment type of the instructor (i.e., human instructor vs. animated instructor) on learner’s visual attention, neural activity, and learning outcome.

Few studies have investigated the student’s neural activity during multimedia learning. An earlier electroencephalography (EEG) study (13) revealed decreased theta band power implying lower cognitive load, reduced self-reported cognitive effort, and improved learning performance with an onscreen instructor compared with no onscreen instructor. An functional near-infrared spectroscopy (fNIRS) study (14) tested whether animated anthropomorphic pedagogical agents could present social cues that elicit neural responses in the mentalizing network engaged in mentalizing and social reasoning (15). The authors found improved learning performance, less self-reported cognitive load, and increased neural activation in the mentalizing network due to such pedagogical agents. Such findings suggest that we can systematically examine from a neurocognitive perspective whether there is a direct relationship among instructor’s image, cognitive load, and socio-emotional processing of the learner, and if there is, how it is related to the learning outcome.

In this study, we use a data-driven, intersubject correlation (ISC) analysis (16–18), to study the neural mechanisms in multimedia learning, specifically the joint contributions of neural networks implicated in attentional control, socio-emotional processing, and working memory according to the extant neuroimaging literature (19–22). ISC has been a useful paradigm in naturalistic neuroimaging studies, especially when participants receive audiovisual materials that are continuously presented (e.g., instructional videos) while their neural responses are recorded (23–26). Robust ISC was consistently found not only in the perceptual cortex but also in higher-order brain regions associated with language comprehension, cognitive control, mentalizing, and learning (23, 25–28). In our study, ISC is used to measure shared stimulus-locked responses by correlating the time series of BOLD activity across subjects for individual voxels or regions to isolate stimulus-driven neural synchronization during multimedia learning. Furthermore, recent studies (29, 30) have applied ISC analysis to the moment-to-moment eye-movement data during video learning, showing that ISC can be predictive of video learning performance when it is derived from eye gaze positions (29) and such ISC is also correlated with EEG-based brain synchrony data (30). In this study, we further extend the methodology to a combined dataset from eye movements and functional MRI data to analyze how learners allocate visual attention and process audiovisual and social-cognitive information during video learning.

To further examine individual differences underlying multimedia learning, we used “intersubject representational similarity analysis” (IS-RSA) (28, 31), a method that uses second-order similarity akin to representational similarity analysis (32). It compares subject-by-subject pairwise brain-based ISC matrices and subject-by-subject pairwise behavioral individual difference matrices. IS-RSA can thus reveal links between individual differences in brain activity and behavior and offer insights into the neural underpinnings of unique stimuli characteristics. In a recent study (26), this method was used to demonstrate that measures of social desire and self-control correlate with brain networks in participants during the viewing of only emotion-laden videos, but not neutral videos. Using IS-RSA, we also aim at further discerning varying levels of processing demands imposed by video learning formats (e.g., with the presence or absence of onscreen instructors). Previous research (33) provided evidence that individuals with different working memory abilities have different learning outcomes when facing elevated cognitive load (e.g., language learning in virtual reality). We hypothesized that, under heightened processing demands, participants with higher abilities will likely converge on similar cognitive strategies and thus show more synchronized neural activities. Such convergence or alignment would manifest as positive correlations between behavioral data and neural data, for example, individuals with higher abilities show higher similarity when IS-RSA is applied to both sets of data. If such correspondences are observed under one but not the other condition, it would suggest that one condition imposes a specific processing demand on the learner in comparison to the other (26).

**Fig. 1.**
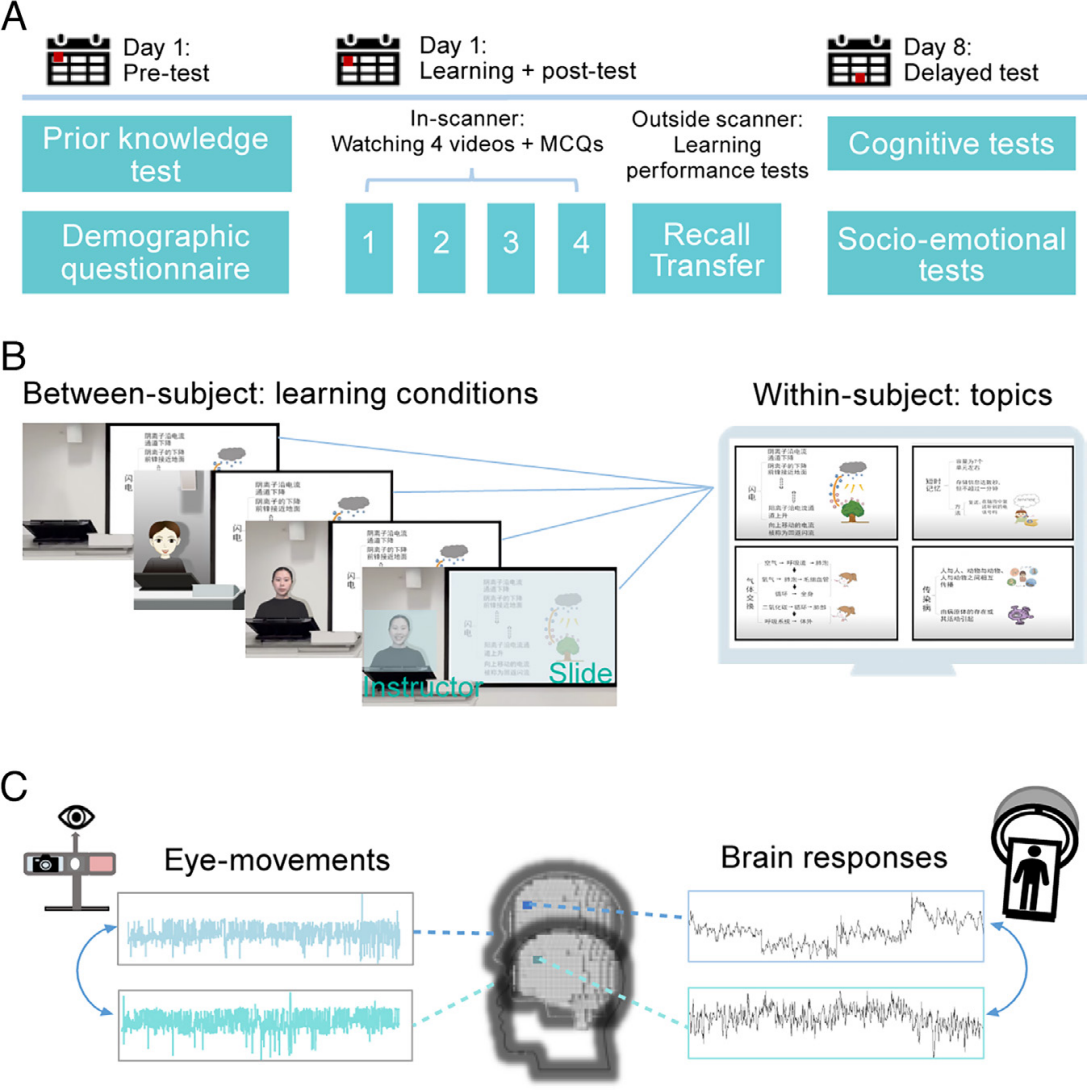
A schematic diagram of experimental procedure and data analysis. (*A*) The experimental procedure. Participants took prior knowledge tests and filled in their demographic information before the main experiments. During the main experiment, which took place in the MRI scanner, participants watched video lectures and completed multiple comprehension questions (MCQs) that tap into their comprehension of the learning content. They were then sent out of the scanner to complete recall and transfer questions. During the delayed test, participants completed cognitive or sociability/emotionality tests (*Behavioral Measurements*). (*B*) The fMRI experimental design. Each participant watched four lectures on videos covering four different topics (within-subject), under one of the four learning conditions (between-subjects). We constructed two rectangular AOIs enclosing the instructor face and the content slides, respectively. (*C*) ISC analysis applied to both eye movements and fMRI signals, which were simultaneously recorded during video watching.

Taken together, the current study sets out to use ISC and IS-RSA to examine multimodal data obtained from naturalistic neuroimaging with simultaneous recordings of participants’ functional magnetic resonance imaging (fMRI), eye movements, learning performance, and their individual cognitive and socio-emotional processing abilities (Fig. 1). First, we ask whether students’ learning performance will benefit from the presence of the instructor’s image, thereby testing the image principle, and whether a higher level of humanness would engage stronger socio-emotional processing in multimedia learning, thereby testing the embodiment principle. Second, we examine the underlying neural correlates of the onscreen presence of the instructor and the humanness level of instructor and ask how neural patterns may reflect cognitive and socio-emotional processing. Third, by linking our multimodal behavioral and neuroimaging data, we examine what costs or benefits the onscreen instructor may have for the learner that give rise to either improved or impoverished learning performance. We predict that the onscreen presence of an instructor, especially a human instructor (as compared with the animated instructor), would attract the learner’s attention more strongly during learning. This may lead to deeper cognitive processing even when learners devote less attention to the learning content on the slide. We also expect that if the onscreen instructor sufficiently motivates learners, it may lead to higher levels of eye-movement synchronization and neural synchronization and their second-order correspondence, indicative of stronger cognitive engagement and socio-emotional processing during learning. Such alignment could also further correlate with the learning outcome.

## Results

### Students Learned Better with an Onscreen Instructor but Learning Was Not Affected by whether the Instructor Was Human or Animated.

**Fig. 2.**
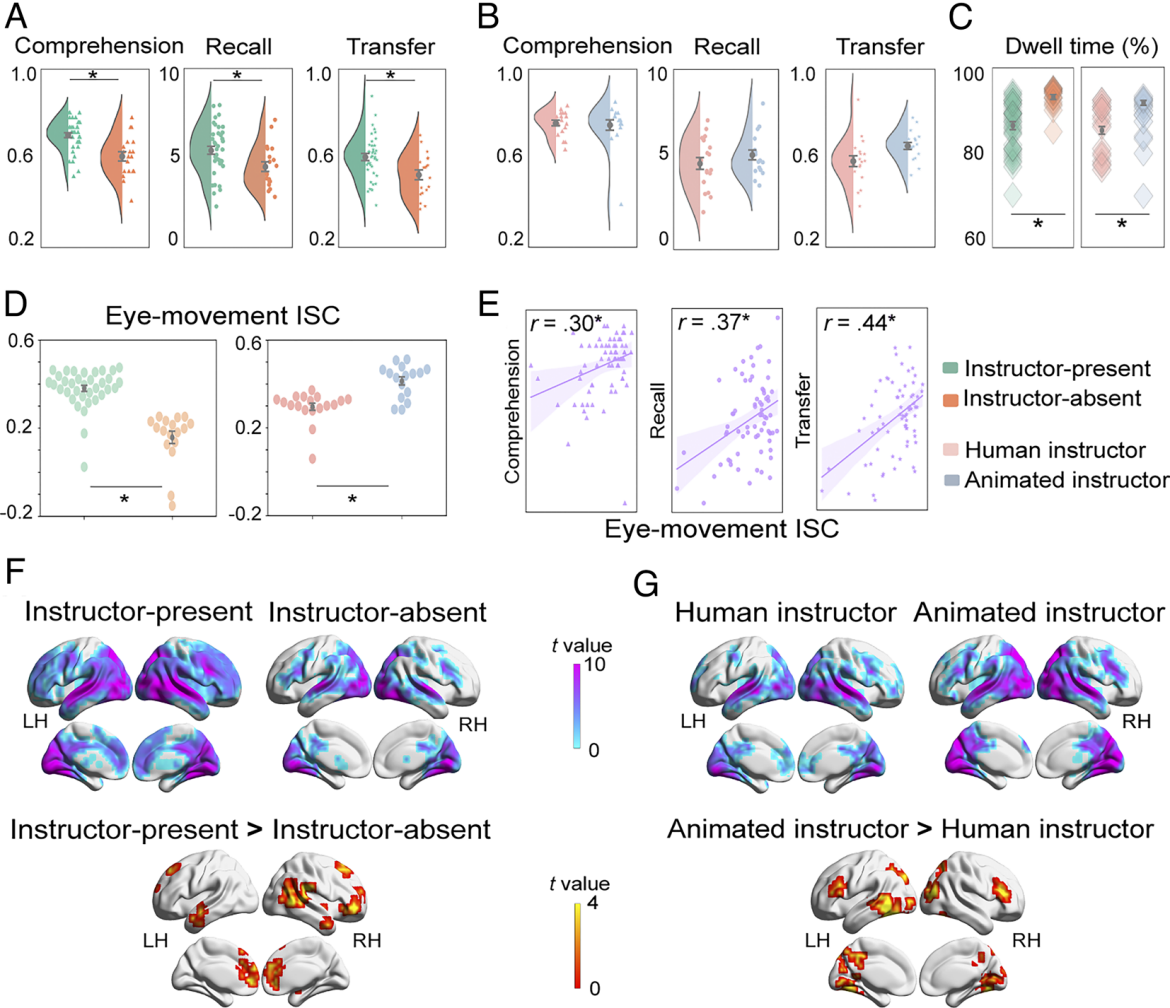
Learning performance, eye fixation duration, and eye-movement and brain ISC results for the image and embodiment principles. (*A*) Effect of onscreen presence of instructors on recall, comprehension, and transfer scores. The instructor-present learners (N = 38) significantly outperformed the instructor-absent learners (N = 19) across all three learning performance measurements (*p*s < 0.05). (*B*) Effect of the embodiment type of instructor (human, N = 18 vs. animated, N = 16) on recall, comprehension and transfer scores (NS; *p*s > 0.05). (*C*) Effect of instructor presence and instructor embodiment type on eye fixations on the lecture slides. Learners allocated less eye fixation on the lecture slides indicated by significantly lower dwell time percentage on the slide AOI, under the instructor-present condition (*P* < 0.05) and under the human instructor condition (*P* < 0.05). (*D*) Effect of instructor presence and instructor embodiment type on eye movement synchronization. The instructor-present condition as compared with the instructor-absent condition, and the animated instructor condition as compared with the human instructor condition, both resulted in more synchronized eye movements (*P* < 0.05). (*E*) Correlations between eye-movement ISC and learning performance. More synchronized eye movements were positively correlated with better learning performance scores concatenated across all three learning conditions (*p*s < 0.05). (*F*) Effect of instructor presence on learners’ neural synchronization. The instructor-present condition elicited significantly greater neural synchronizations than the instructor-absent condition. (*G*) Effect of the embodiment type of the instructor on learners’ neural synchronization. The animated instructor condition elicited significantly greater neural synchronizations than the human instructor condition. Notes: Asterisks in the plots indicate statistically significant results (i.e., *P* < 0.05), and error bars indicate the SEs.

The behavioral data comprised the subjects’ scores on the comprehension, recall, and transfer tests (*Materials and Methods*) under different learning conditions. First, we tested the image principle by determining whether the social presence of the instructor would hinder or improve learning performance. Specifically, we compared learning performance under the instructor-present (N = 38) and instructor-absent (N = 19) conditions. The former condition included both the instructor-present/neutral human (N = 20) and instructor-present/smiling human (N = 18) sub-groups, given that no significant difference was observed between the two conditions in both behavioral and neural domains (See *SI Appendix,* Fig. S1 and Tables S1 and S4; all reports below were based on analyses of the aggregated data of these two conditions). We conducted three ANCOVA tests and found that learners under the instructor-present condition obtained significantly higher comprehension (*F*_1,50_ = 18.78, *P* < 0.001, *η_p_^2^* = 0.27), recall (*F*_1,50_ = 4.99, *P* = 0.030, *η_p_^2^* = 0.09), and transfer scores (*F*_1,50_ = 6.83, *P* = 0.012, *η_p_^2^* = 0.12) than learners under the instructor-absent condition (Fig. 2*A*). These results indicate that students learned better with an onscreen instructor.

The second analysis was to determine whether the embodiment type of the instructor would affect learning performance, thus testing the embodiment principle. Specifically, we compared learning performance under the human instructor (N = 18) and animated instructor (N = 16) conditions.^*^ Similarly, we adopted three ANCOVA tests. Fig. 2*B* shows no significant differences between the conditions in comprehension (*F*_1,27_ = 0.03, *P* = 0.87, *η_p_^2^* = 0.001), recall (*F*_1,27_ = 0.87, *P* = 0.36, *η_p_^2^* = 0.03), or transfer test scores (*F*_1,27_ = 2.16, *P* = 0.15, *η_p_^2^* = 0.07). Thus, students learned at equivalent levels for both types of onscreen instructors (human or animated).

### Students Attended More to the Slides When an Instructor Was Not Present Onscreen or When the Instructor Was Animated.

Following the standard in the multimedia learning literature (34), we extracted four fixation-related measurements on the slide’s area of interest (AOI; Fig. 1*B*) under the instructor-present and instructor-absent conditions, respectively, to examine the learner’s attention allocation during learning (*Materials and Methods*). Four ANCOVA tests were performed to examine the image principle by determining the effect of instructor presence on learners’ attention allocation. Results revealed that dwell time percentage (*F*_1,50_ = 27.92, *P* < 0.001, *η_p_^2^* = 0.36; see Fig. 2*C*) and fixation count percentage on the slides (*F*_1,50_ = 16.32, *P* < 0.001, *η_p_^2^* = 0.25) were both larger under the instructor-absent condition than the instructor-present condition. For other measurements, there were no significant differences between these two conditions (dwell time: *F*_1,50_ = 0.08, *P* = 0.77, *η_p_^2^* = 0.002; fixation count: *F*_1,50_ = 0.38, *P* = 0.54, *η_p_^2^* = 0.008). In summary, compared with the instructor-absent condition, learners under the instructor-present condition fixated less on slides, suggesting less attention devoted to the learning content due to the onscreen presence of the instructor.

We further extracted four fixation-related measurements for the AOIs on the slide and on the instructor’s image, respectively, under both the human instructor and the animated instructor conditions (see Fig. 1*B* for the AOIs). Eight ANCOVA tests were implemented to discern the effect of the instructor’s embodiment type on these eye fixation-related measurements. Fig. 2*C* showed that learners under the animated instructor condition, compared with the human instructor condition, spent a significantly larger portion of dwell time on the learning content (*F*_1,27_ = 9.10, *P* = 0.006, *η_p_^2^* = 0.25), while there was no significant difference in other measurements (dwell time: *F*_1,27_ = 0.78, *P* = 0.38, *η_p_^2^* = 0.028; fixation count percentage: *F*_1,27_ = 3.40, *P* = 0.08, *η_p_^2^* = 0.11; fixation count: *F*_1,27_ = 0.69, *P* = 0.41, *η_p_^2^* = 0.03). Learners also devoted significantly lower dwell time percentage (*F*_1,27_ = 11.14, *P* = 0.002, *η_p_^2^* = 0.29), shorter dwell time (*F*_1,27_ = 8.60, *P* = 0.007, *η_p_^2^* = 0.24), lower fixation count percentage (*F*_1,27_ = 4.99, *P* = 0.034, *η_p_^2^* = 0.16), and fewer fixation counts (*F*_1,27_ = 7.30, *P* = 0.012, *η_p_^2^* = 0.21) on the animated instructor than on the human instructor, suggesting that learners attended less to the slides when the instructor was human than when it was animated.

### More Synchronized Eye Movements Were Correlated with Better Learning.

In order to quantify eye movement synchronization under different learning conditions, we derived eye-movement ISC scores for each learner using the moment-to-moment gaze positions while viewing the videos (*Materials and Methods* and Fig. 1*C*). We conducted two analysis of covariance (ANCOVA) tests to examine under which condition eye movements during video learning were more synchronized across learners. Next, we correlated individual eye-movement ISC scores with individual learning performance under each condition. Finally, we concatenated eye-movement ISC scores and learning performance scores across conditions to test whether there was an overall correlation between synchronized eye movements and learning performance.

We found that learners under the instructor-present condition showed significantly higher eye-movement ISC scores than learners under the instructor-absent condition (*F*_1,47_ = 59.59, *P* < 0.001, *η_p_^2^* = 0.53; Fig. 2*D*). Learners under the animated instructor condition also showed significantly higher ISC scores than those under the human instructor condition (*F*_1,27_ = 27.88, *P* < 0.001, *η_p_^2^* = 0.43). Further, eye-movement ISC scores were positively correlated with comprehension scores under the instructor-present (*r* = 0.33, *P* = 0.023; Fig. 2*E*) and under the human instructor (*r* = 0.42, *P* = 0.042) conditions. After concatenating eye-movement ISC scores across all conditions, we observed a condition-independent positive correlation between eye-movement ISC and learning performance (recall: *r* = 0.37, *P* < 0.001; transfer: *r* = 0.44, *P* < 0.001; comprehension: *r* = 0.30, *P* = 0.007). These results indicated that individual eye-movement ISC was predictive of learning performance, suggesting that learners who allocate their visual attention more similarly are those who learn better, consistent with other recent eye-movement findings (29).

### Greater Neural Synchronizations Were Observed When the Instructor Was Present or When the Instructor Was Animated.

To assess the degree of neural synchronization across learners under instructor-present and instructor-absent learning conditions, we constructed two group-level ISC maps (*Materials and Methods* and Fig. 1*C*). We found significant ISCs across a large range of the visual, auditory, and higher-order cortical regions, including superior temporal gyrus (STG), middle temporal gyrus (MTG), dorsomedial prefrontal cortex, and posterior parietal cortex (Fig. 2*F*) under both conditions. Next, we tested the image principle by examining brain regions that had different synchronization patterns under the two conditions. The results suggested that relative to the instructor-absent condition, the instructor-present condition evoked significantly greater neural synchronizations in the left fusiform gyrus (FG) and anterior cingulate cortex (ACC), the right mPFC, STG, MTG, and superior frontal gyrus (SFG; Fig. 2*F* and *SI Appendix*, Table S5). It is worth noting that FG is a classical region specialized in face processing (35), and higher ISC in this region indicates that the onscreen instructor’s image led to secondary processing not directly related to the learning content. For the human and animated instructor conditions, there were significant ISCs in visual and auditory regions, high-order language regions, and frontoparietal executive control regions (Fig. 2*G*). Further, with regard to the embodiment principle, greater neural synchronizations were elicited in more brain regions by the animated instructor condition than by the human instructor condition in visual and higher-order regions (Fig. 2*G* and *SI Appendix*, Table S5) including the right FG, middle occipital gyrus, inferior frontal gyrus (IFG), the left LG, MTG, MFG, and superior parietal lobule (SPL).

The above results suggest neural synchronization emerges across all learning conditions in key cognitive brain regions. Greater neural synchronization under the instructor-present (as compared with the instructor-absent) condition illustrated a higher degree of neural synchrony induced by the onscreen presence of the instructor. However, a lower degree of neural synchronization was identified under the human instructor condition in several brain regions, suggesting greater variability in neural responses induced by the human (as compared with the animated) instructor. These ISC patterns are consistent with those from the eye-movement ISC analysis, for both comparisons. We return to the interpretation of this finding in *Discussion*.

### Neural Synchrony among Higher-Ability Learners Entailed Additional Cognitive and Socio-Emotional Processing Demands from the Onscreen Instructor.

Using the IS-RSA method (28, 31), we constructed pairwise behavioral similarity matrices based on learners’ cognitive and socio-emotional scores where higher pairwise similarity was generated among individuals with higher scores (see *Materials and Methods* and *SI Appendix*, S6 for details) and then searched for brain regions where the derived brain ISC matrices exhibit positive correlations with the behavioral matrices. By doing this analysis, first, we wanted to pinpoint brain regions where neural ISC matrices are similarly structured as behavioral matrices, so as to identify the brain region’s involvement in the relevant cognitive processes (28, 31, 32). Second, we also wanted to see whether a brain–behavior correlation exclusive to one condition exists, and if so, how it entails unique processing demands of this condition as compared with another condition (26). Following previous literature using IS-RSA (28), we hypothesize that given higher processing demands, those learners with higher abilities tend to exhibit more homogeneous neurocognitive strategies to tackle the increased demands, manifested as synchronized brain activity. This hypothesis provides the basis for the positive correlations between the neural and behavioral matrices.

**Fig. 3.**
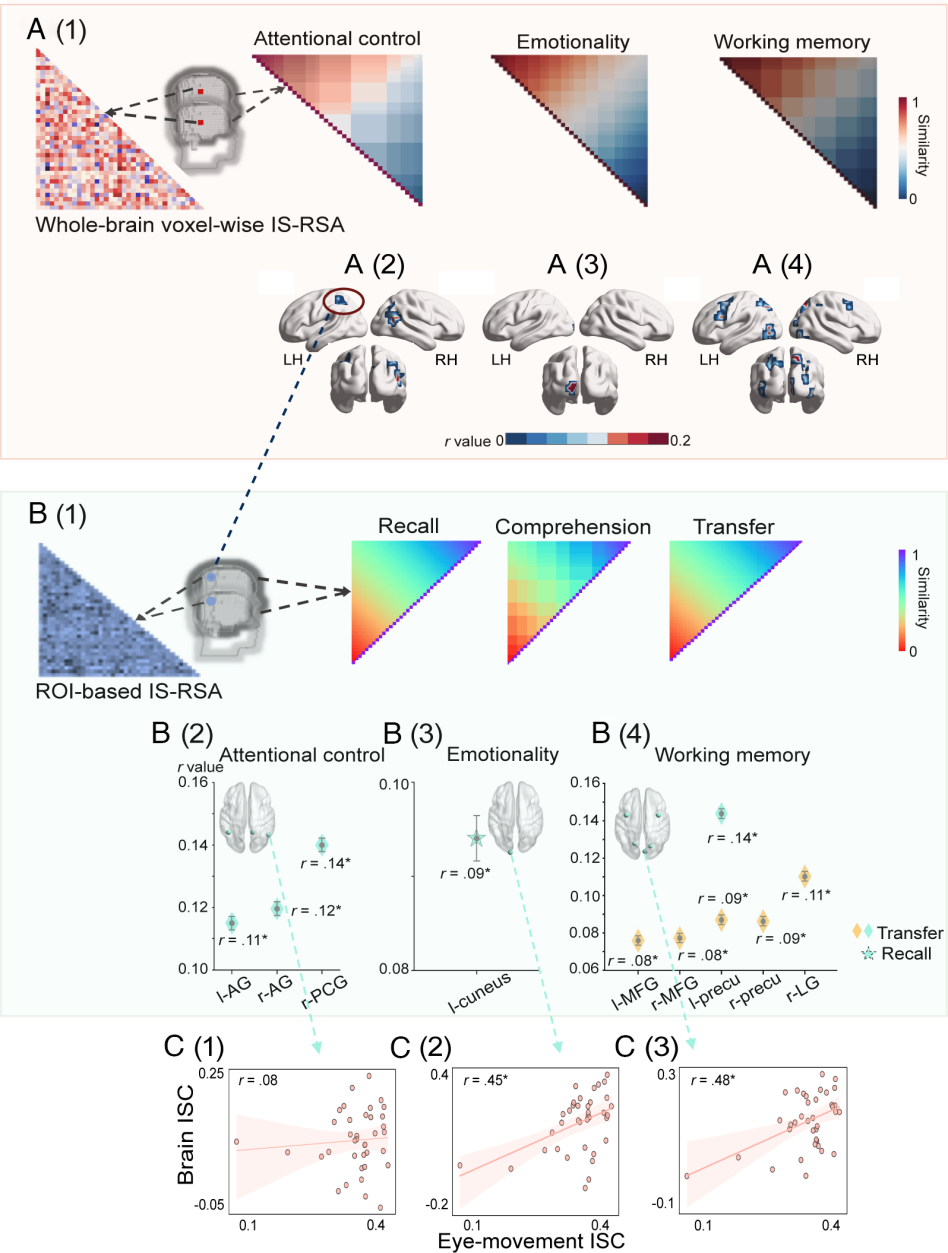
Intersubject representational similarity analysis (IS-RSA) and eye–brain correlational analysis for the image principle. (*A*) whole-brain voxel-wise IS-RSA results for the instructor-present learning condition (N = 38). [*A*(1)] is an illustration of the whole-brain IS-RSA: three pairwise matrices based on learners’ pairwise attentional control, emotionality, and working memory scores are shown to be significantly correlated with intersubject neural responses in key brain regions, including right PCG and bilateral AG implicated in conflict monitoring during learning [*A*(2)], the left cuneus involved in emotional processing [*A*(3)], right LG, the left IFG, bilateral precuneus, and bilateral MFG involved in working memory-related processes [*A*(4)]. (*B*) ROI-based IS-RSA results for the instructor-present learning condition: three pairwise matrices based on learners’ recall, comprehension, and transfer scores were further correlated with neural synchronization in ROIs implicated in the above three cognitive/socio-emotional processes [*B*(1)], including bilateral AG and the right PCG [*B*(2)], regions implicated in attentional control, which were positively correlated with the transfer score matrix where higher scores generated higher similarity (blue diamonds); left cuneus [*B*(3)], a region implicated in socio-emotional processing, which was also positively correlated with learners’ recall performance with similar scores showing higher similarity (blue star). Among the brain regions involved in working memory processing, bilateral MFG, bilateral precuneus, and the right LG [*B*(4)] showed significant correlation with learners’ transfer score matrix where similar scores showing higher similarity (yellow diamonds). Neural synchronization in the left precuneus [*B*(4)] was further correlated with the transfer score matrix where higher scores generated higher similarity (blue diamond). All *P* values were corrected for multiple comparisons to control FDR at *P* < 0.05. (*C*) Eye–brain correlation analysis (N = 37). Individual eye-movement ISC was not correlated with brain regions implicated in attentional control [e.g., right AG; *C*(1)]. Individual eye-movement ISC was correlated with individual brain ISC in the left cuneus involved in socio-emotional processing [*C*(2)] and the right LG related to working memory [*C*(3)]. Notes: The left and right brain regions were marked with “l-” and “r-” respectively; asterisks indicate statistically significant results (i.e., *P* < 0.05); Error bars in [*B*(2), *B*(3), and *B*(4)] indicate the SEs of bootstrapped correlational coefficients.

Fig. 3*A* showed that during instructor-present multimedia learning, the behavioral matrix grounded on attentional control was positively correlated with brain ISC matrices in the right posterior cingulate gyrus (PCG; *r* = 0.21, *P* < 0.05) and bilateral angular gyrus (AG; left: *r* = 0.17, *P* < 0.05; right: *r* = 0.21, *P* < 0.05). Individual variations in socio-emotional processing indexed by emotionality scores were also correlated with brain ISC matrices in the left cuneus (*r* = 0.37, *P* < 0.05). Finally, working memory matrices were positively correlated with brain ISC matrices in bilateral precuneus (left: *r* = 0.17, *P* < 0.05; right: *r* = 0.22, *P* < 0.05), bilateral MFG (left: *r* = 0.18, *P* < 0.05; right: *r* = 0.20, *P* < 0.05), left IFG (*r* = 0.17, *P* < 0.05), as well as in right early visual cortex/LG (*r* = 0.26, *P* < 0.05). In contrast to the significant correlations under the instructor-present condition, for the instructor-absent condition, we did not find any significant positive correlations or alignment between matrices based on behavioral scores and brain ISC. These findings suggest that the onscreen presence (as compared with the absence) of an instructor could be more taxing on cognitive and socio-emotional processing. Under the instructor-present condition, participants with higher attentional control, working memory, and emotionality showed more synchronized neural responses in coping with such elevated demands, giving rise to the significant behavior–brain correlations.

**Fig. 4.**
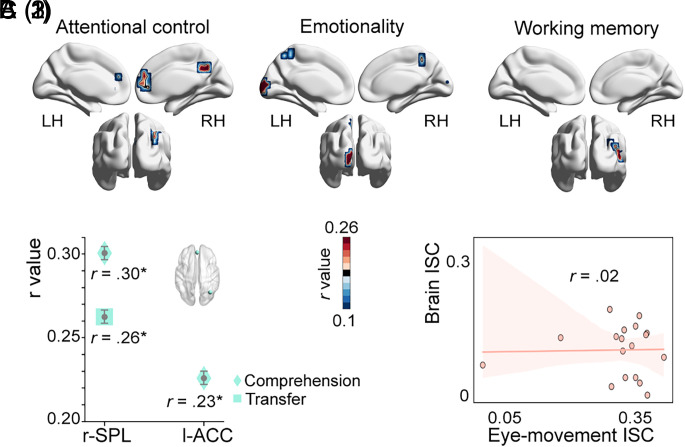
Intersubject representational similarity analysis (IS-RSA) and eye–brain correlational analysis for the embodiment principle. (*A*) Whole-brain IS-RSA results (N = 18): [*A*(1)] shows that right SPL and precuneus and the left ACC were involved in attentional control during learning; [*A*(2)] shows that the left cuneus and precuneus were involved in socio-emotional processing, and [*A*(3)] shows that right MTG was engaged in working memory. (*B*) ROI-based IS-RSA results. Only the right SPL and the left ACC implicated in attentional control showed neural synchronization significantly correlated with pairwise comprehension and transfer score matrices where higher scores generated higher similarity. (*C*) Eye–brain correlational analysis. Individual eye-movement ISC was not significantly correlated with individual brain ISC in the three brain regions in *A*(1) implicated in attentional control. The correlational pattern in the (*C*) panel is based on an example from the left ACC, to illustrate the lack of correlation between eye-movement ISC and brain ISC. Notes: Asterisks indicate statistically significant results (i.e., *P* < 0.05); error bars in (*B*) indicate the SEs of bootstrapped correlational coefficients.

We further performed the same whole-brain IS-RSA focused on only the human instructor and animated instructor learning conditions in order to examine the embodiment principle. As shown in Fig. 4*A*, first, intersubject variations in attentional control were positively correlated with ISC matrices in the right superior parietal lobule (SPL; *r* = 0.29, *P* < 0.05) and precuneus (*r* = 0.38, *P* < 0.05), and the left ACC (*r* = 0.38, *P* < 0.05). Next, intersubject variations were positively correlated with ISC matrices in the left cuneus (*r* = 0.60, *P* < 0.05) and precuneus (*r* = 0.24, *P* < 0.05) for emotionality, and in the right MTG for working memory (*r* = 0.34, *P* < 0.05). These results showed that higher neural synchronization under the human instructor condition occurred for learners with higher abilities in attentional control, emotionality, and working memory. In contrast, no such correlations were observed under the animated instructor condition.

### Neural Synchrony in Cognitive and Socio-Emotional Processing Was Associated with Learning Performance.

The above IS-RSA allowed us to identify key brain regions implicated in attentional control, socio-emotional processing, and working memory during multimedia learning with an onscreen instructor. We further conducted ROI-based IS-RSAs to identify whether brain activity in specific regions involved in such processes might be correlated with specific learning performance. Based on previous neuroimaging work of individual difference (27), we hypothesized that those with either higher or similar behavioral performance may exhibit more similar neural responses, giving rise to higher neural synchrony (*Materials and Methods* and *SI Appendix*, S6).

As shown in Fig. 3*B*, first, both bilateral AG (left: *r* = 0.11, *P* < 0.05; right: *r* = 0.12, *P* < 0.05) and the right PCG (*r* = 0.14, *P* < 0.05), regions implicated in attentional control, were positively correlated with the transfer score matrix where higher scores engendered higher pairwise similarity. In addition, the left cuneus (*r* = 0.09, *P* < 0.05), involved in socio-emotional processing, was positively correlated with learners’ recall performance with similar scores generating higher pairwise similarity. Among the brain regions involved in working memory, the left precuneus (*r* = 0.14, *P* < 0.05) was further correlated with the transfer score matrix where higher transfer scores generated higher pairwise similarity; concurrently for other brain regions involved in working memory, including bilateral MFG (left: *r* = 0.08, *P* < 0.05; right: *r* = 0.08, *P* < 0.05), bilateral precuneus (left: *r* = 0.09, *P* < 0.05; right: *r* = 0.09, *P* < 0.05), and the right LG (*r* = 0.11, *P* < 0.05), they showed significant correlation with learners’ transfer score matrix with similar transfer scores generating higher similarity. These correlations of specific learning performance with specific brain regions suggest the engagement of the relevant cognitive and socio-emotional processing components in multimedia learning.

We further similarly performed ROI-based IS-RSA for the human instructor condition. Fig. 4*B* shows that the right SPL related to attentional control was positively correlated with comprehension (*r* = 0.30, *P* < 0.05; see Fig. 4*B*) and transfer (*r* = 0.26, *P* < 0.05) matrices where higher scores generated higher similarity. The left ACC, also related to attentional control, showed significant correlation with comprehension matrices constructed similarly (*r* = 0.23, *P* < 0.05). However, unlike in the instructor present-vs.-absent conditions, neural synchronization here was not significantly correlated with learning performance for regions implicated in socio-emotional and working memory processing. We also found that those who gained better learning performance did not allocate significantly more visual attention to the human instructor (dwell time: *t*(16) = −0.66, *P* = 0.52, *d* = 0.31; fixation count: *t*(16) = −0.07, *P* = 0.95, *d* = 0.03). This contrasted with the animated instructor condition, where those with higher learning performance fixated significantly more on the animated instructor (dwell time: *t*(14) = 2.59, *P* < 0.05, *d* = 1.29; fixation count: *t*(14) = 3.30, *P* < 0.01, *d* = 1.65). Interestingly, the human instructor and animated instructor conditions yielded comparable learning performance, as mentioned earlier. The fact that the different amount of attention to the instructor’s image did not lead to different learning outcomes under human vs. animated instructor conditions provides an important insight for understanding the effects of cognitive and socio-emotional processing and visual attention, to which we return in *Discussion*.

### Eye–Brain–Learning Correspondence Revealed Critical Roles of Socio-Emotional Processing and Working Memory.

Given the findings above, we attempted to connect eye movement, brain activity, and learning outcome under a unified framework. We first correlated individual eye-movement ISC with individual brain ISC for each ROI where there was a significant correlation between neural synchronization and learning performance. In other words, these eye–brain correlation analyses were done only when the brain ISC was predictive of learning performance. The results showed that in the instructor-present condition, individual learners’ brain ISC obtained from regions related to socio-emotional processing (i.e., the left cuneus) and working memory (i.e., the right precuneus and LG) were significantly correlated with individuals’ eye-movement ISC (cuneus: *r* = 0.45, *P* < 0.01; precuneus: *r* = 0.36, *P* < 0.05; LG: *r* = 0.48, *P* < 0.01; see Fig. 3*C*). However, individual brain ISC scores in regions related to attentional control (e.g., ACC) were not correlated with eye-movement ISC (*p*s > 0.05; see Fig. 3*C*). Next, we derived eye-movement ISC matrices and correlated them with brain ISC matrices in each ROIs (i.e., eye movement-brain IS-RSA). Consistent with the individual ISC patterns, the IS-RSA results indicated that the neural synchrony patterns were positively correlated with the eye-movement synchronization patterns in the left cuneus (*r* = 0.29, *P* < 0.01) and right LG (*r* = 0.21, *P* = 0.056), two regions respectively implicated in socio-emotional processing and working memory, but not in ACC or other attentional control-related regions. Under the human-instructor condition, we also performed the same individual brain and IS-RSAs between neural synchronization and eye-movement synchronization. As with the instructor-present condition, neural synchronization in attentional control-related brain regions was not correlated with eye-movement synchronization (Fig. 4*C*). Thus, our eye–brain–learning correspondence data revealed the important roles of socio-emotional processing and working memory in multimedia learning.

## Discussion

The current study adopted a naturalistic multimodal neuroimaging approach in order to capture visual attention allocation and neurocognitive patterns underlying multimedia learning. Toward this goal, we collected behavioral, eye-movement, fMRI, and learning performance data from student learners in an instructional video learning context. We further relied on data analytics to identify behavioral and neural synchrony patterns, individual differences in cognitive and socio-emotional processing, and their relationships with learning outcome. Our findings can be summarized as follows. First, learners allocated a substantial amount of attention to the onscreen instructor, especially the human instructor, which did not hinder the learning outcome (contrary to some previous studies). Second, learners exhibited more synchronized eye movements under the instructor-present condition and also the animated instructor condition, which were associated with better learning outcomes. Third, the instructor’s social presence (both human and animated instructors) elicited higher neural synchronization in regions related to cognitive and socio-emotional processing. Fourth, behavior–brain analyses showed that the onscreen presence of an instructor, especially human instructor, led to simultaneously heightened attentional control, more in-depth socio-emotional processing, and deeper cognitive processing. Finally, our eye–brain–learning correspondence analyses revealed that the neural synchronization and eye-movement synchronization patterns were aligned in brain regions involved in socio-emotional processing and working memory when there was onscreen presence of an instructor (as opposed to when there was none), which was also predictive of the learning outcome.

These findings allowed us to better understand the debates in the literature revolving around two key principles in the multimedia learning theory: the image principle and the embodiment principle (3, 4). Extant literature has focused on whether the instructor’s image would cause distraction to the learners. However, our data showed that while the instructor’s image can be distracting (i.e., diverting attention from the learning content), the onscreen social presence of the instructor would motivate students to learn, especially those students with higher cognitive and socio-emotional abilities. We contend that the instructor’s image enables them to engage in deeper cognitive processing, therefore neutralizing or diminishing the negative effect of distraction. However, when such socio-emotional cues cannot outweigh the distraction caused by the instructor’s presence, the benefits of onscreen instructor’s social presence may not be readily accessible.

### Instructor Image Engages More Synchronized Eye Movements, Additional Attentional Control, Deeper Socio-Emotional Processing, and Greater Working Memory.

Previous eye-tracking studies indicated that learners allocate substantial attention to the instructor’s image (up to 27% of their total visual attention during video learning) (10–12). Some studies showed a null or even negative effect of the instructor’s onscreen presence on learning outcomes (11, 12, 36) while others found an advantage of the instructor’s presence (13, 37). Our study indicated that, compared to previous studies, our learners allocated a smaller proportion of visual attention to the instructor’s image. However, our data showed that the learners with onscreen instructors had a higher degree of synchronized eye movements and better learning outcomes, and significantly and unequivocally outperformed the learners without onscreen presence of the instructor.

Consistent with the eye-movement findings, the neuroimaging findings also showed key brain regions with significantly greater ISCs under the instructor-present condition than the instructor-absent condition. First, the right mPFC, a key component of the mentalizing network (20, 38), exhibited greater neural synchronization across learners. This also held true for the right STG and MTG, in line with other ISC studies using audiovisual stimuli (25, 27, 39) and with an fNIRs study (14) pinpointing the joint role of STG and MTG in social interaction and mentalizing during multimedia learning. Second, under the instructor-present condition, we also identified greater ISCs in the left ACC in the ventral attention network (19, 40) and the right SFG crucial for working memory (40). Third, FG, a core facial perception region (35, 41), showed greater neural synchronization which, as mentioned earlier, indicates that the social presence of the instructor led the learner to perform secondary processing not related to learning (i.e., face processing). Nevertheless, this additional social processing did not impede but enhanced learning. Given this finding, we suggest that the instructor’s image not only evokes extra demand for attentional control but also conveys positive socio-emotional and cognitive benefits. Better learning performance is only possible if learners are sufficiently motivated by the instructor’s onscreen presence to have more in-depth cognitive processing.

Our whole-brain IS-RSAs further identified brain regions where neural synchrony correlates with behavioral task scores in attentional control, socio-emotional processing, and working memory, especially among those with higher abilities. These analyses showed that the same conditions of multimedia learning may engage different learners in different ways. Specifically, the bilateral AG and PCG exhibited greater synchronization across participants with higher attentional control abilities when there was instructor’s onscreen presence. AG has been considered a hub of the default mode network (42) and the attention network. PCG is also a core region of the DMN, serving a central role in internally directed cognition (42, 43). The involvement of these regions points to the processes of attentional regulation and attentional control during learning given the social presence of instructor. Other key regions in the mentalizing and emotional processing networks (e.g., cuneus) (41, 44) and in working memory (e.g., IFG, MFG, and LG) (45–49) were also engaged under the instructor-present condition, suggesting that these brain regions were recruited for greater socio-emotional and deeper cognitive processing. Overall, these findings suggest that video learning with the onscreen presence of an instructor entails extra cognitive and socio-emotional processing demands, and learners with higher abilities showed higher consistency in brain and behavioral patterns when coping with such extra processing demands.

### Stronger Socio-Emotional and Cognitive Processing Is Related to Eye Movement Synchronization and Learning Performance.

Given our findings, one may wonder whether the onscreen instructor’s social presence is related not only to more cognitive and socio-emotional processing but also better learning outcome. Our study showed a) positive correlation between neural synchronization in the left cuneus involved in socio-emotional processing and learning recall scores and also b) positive correlations between neural synchronization in regions implicated in working memory processing (i.e., bilateral MFG and precuneus and the right LG) and transfer score patterns. Importantly, our eye-movement synchronization data, which positively predicted learning performance, were also shown to be significantly correlated with the above brain regions under the instructor-present condition. These findings jointly underscore the importance of socio-emotional processing and working memory for deeper cognitive processing, a foundation for improved learning outcome in multimedia learning as predicted by social agency theory (3, 4).

Although brain regions involved in attentional control were also significantly correlated with learning performance, unlike (a) and (b), there was no correlation between these regions and learners’ eye movement patterns. This lack of correlation suggests that the role of attentional control for learning outcome is less clear as compared with that of socio-emotional processing and working memory, perhaps because it tends to reflect the distraction caused by the presence of an instructor (i.e., monitoring the instructor’s presence while allocating attention to the learning content). Nevertheless, our eye-movement data, coupled with our neural and learning performance data, are highly aligned with recent findings (29) that the learners who exhibit more synchronized eye movements tend to learn better. The combined eye-movement ISC and brain ISC patterns (and their correlations with each other and with learning performance) motivate our claim that the social presence of the onscreen instructor elicits more synchronized neural activities and eye movements during learning, which is further predictive of better learning performance.

In an effort to assess the unique contributions (or lack thereof) of the brain ISC and eye-movement ISC to learning, we further performed hierarchical regression analyses with two types of models: One model in which eye-movement ISC was included in the first step and brain ISC added in the second step and another model in which brain ISC was included in the first step and then eye-movement ISC added in the second step (*SI Appendix*, S2 and Tables S6 and S7). Results showed that incorporating both eye-movement ISC and brain ISC in the hierarchical models nominally increased the variance explained, but the additional variance in learning performance accounted for was not significantly greater by either the brain ISC or the eye-movement ISC alone, due to the high collinearity between the two types of data. These analyses prompted us to consider treating brain synchronization and eye-movement synchronization as partially overlapping, rather than causally linked, indices in the context of multimedia learning. Both measurements may capture some unique information that contributes to learning, and both are correlated with one another and with students’ learning performance. Therefore, we conjecture that it is cognitive engagement (29, 30) that drives the observed alignment of brain and eye-movement consistency, and the onscreen instructor serving as a social cue has sufficiently motivated the learner to a higher level of socio-emotional processing and working memory engagement. This occurs despite the learner’s involvement in secondary tasks (processing the instructor’s visual/facial features). Learners under the instructor-present condition may follow the visual content in the video more closely, allocate attention more proactively or switch attention more efficiently, and ultimately achieve better learning outcome.

Previous studies have separately examined learning performance, eye movements, and brain responses (10, 14, 50) and focused on either the instructor presence’s distracting (cognitively taxing) or its motivating (socio-emotional engagement) impacts on learning performance. In contrast, this study integrates multimodal data to examine increased attentional control, socio-emotional processing, working memory engagement, and their relationship with learning performance. Our findings thus help to disentangle the motivational vs. distractive facets of the instructor’s social presence that contribute to students’ learning successes (or failures) and to account for previous mixed findings.

### Embodiment Type of the Onscreen Instructor Influences Cognitive and Socio-Emotional Processing but Not Necessarily Learning Outcome.

In line with previous studies (51, 52), our learners had more eye fixations on the high-embodiment (human) instructor than on the low-embodiment (animated) instructor. Social agency theory (3, 4) suggests that human or human-like characteristics are more appealing and emotionally arousing, which may bring about deeper cognitive processing and thus better learning performance. However, our data did not provide clear evidence for the latter part of this statement. In previous studies (51, 52), a high-embodiment instructor was often accompanied by other multimedia learning signals such as pointing and gestures, whereas in our study, the human instructor was not accompanied by any signaling actions, which may have contributed to the discrepancies observed. In our findings, both eye-movement ISC and brain ISC in the animated instructor condition showed more consistency than in the human instructor condition, suggesting that the human instructor, when compared with the animated instructor, may introduce more variability among learners in cognitive processing and visual allocation.

In our whole-brain IS-RSAs, we showed that the social presence of a human instructor leads to stronger (but also more diverse) socio-emotional processing and working memory engagement. In particular, learners with higher emotionality scores displayed greater neural synchronization in the left cuneus and precuneus of the mentalizing network (20, 44). Moreover, we identified a significant correlation between working memory ability and neural synchronization (higher ability, more synchrony) in the right MTG, commonly implicated in language and semantic memory (53, 54), which may be due to increased processing of the learning content. Further, our results revealed that the presence of a human instructor imposes higher attentional control demands than an animated instructor on the learner, which is reflected in more synchronized neural activities in the right SPL and left ACC, two classic attentional control brain regions. The neural synchronization in the right SPL and left ACC was also further correlated with learning performance but did not correlate with eye movement synchronization patterns. Given these findings, we speculate that attentional control may serve more as a conflict monitoring mechanism during learning rather than directly enhance learning outcome.

### Trade-Off between Cognitive Processing and Visual Distraction in Multimedia Learning.

Our findings here regarding the embodiment principle (from comparing human vs. animated instructor conditions) are consistent with those regarding the image principle (from comparing instructor-present vs. instructor-absent conditions). They all point to the role of concurrently increased demands on socio-emotional processing, working memory, and attentional control imposed by the onscreen presence of a human instructor in video learning. Further linking the neural synchrony in regions implicated in these processes with learning performance, we probed into whether the three processes were directly related to learning outcomes. Here is where we see some differences. While we observed alignment between brain ISC for attentional control-related brain regions and learning performance, we did not observe the alignment between brain ISC in these same regions with eye movement synchronization. These findings suggest that heightened attentional control may be a mirror of the increased distraction imposed by the social presence of a human instructor (an index of the cost), rather than a benefit as socio-emotional processing and working memory are to learning performance. This suggestion also aligns with the previous literature (11, 12), indicating that secondary face processing during online video learning may generate extra attentional cost with the onscreen presence of the instructor, and the cost may divert learners from the primary learning task and hence potentially impede learning. In this connection, it is also important to note that while neural synchrony related to socio-emotional processing and working memory is predictive of more synchronized eye movement patterns and also better learning performance for the image principle, the same eye–brain–learning correspondence could not be said for the embodiment principle.

In order to reconcile the seemingly different patterns of eye–brain–learning correspondence in the image principle and the embodiment principle, we propose a trade-off hypothesis, to account for both the costs and benefits of the social presence of the instructor under different conditions, to explain why different learners may have different learning performance under the same conditions, and to understand why different studies in the literature have generated mixed results regarding the positive or negative effects of onscreen instructor. Specifically, we argue that the onscreen presence of an instructor’s image has both benefits (socially engaging) and costs (cognitively distracting), and it will be up to the learner to leverage the benefit and minimize the cost.

To begin with, the human and animated instructors elicited divergent patterns in the learner’s eye fixation on the instructor: When the onscreen instructor was an animated image, higher-achieving learners tended to have more fixations on the instructor’s image than the lower-achieving learners; when it was a human instructor, the two types of learners did not differ. This may be because when the onscreen instructor is human, it engages an elevated level of socio-emotional processing, while at the same time, it also requires a high degree of attentional control to monitor the distraction so that the learner can effectively switch from the instructor’s image to the learning content and vice versa. When the onscreen instructor is animated, however, it generates a milder socio-emotional response due to the instructor image’s anthropomorphic feature (i.e., simulating human but not really human), while at the same time, it also creates a weaker distraction.

This argument is further solidified by an extra comparison of the brain ISCs between the animated instructor and instructor-absent conditions (*SI Appendix*, S3, Fig. S2, and Table S8). This comparison showed that the presence of an animated instructor evoked a certain degree of socio-emotional response in the right STG, a key part of the mentalizing network, but not the robust facial processing in the classical face processing region FG that was seen in the presence of a human instructor. Therefore, on average, the benefits vs. costs of the trade-off could potentially offset each other, leading to similar learning outcomes in the human vs. the animated instructor conditions. The essence of the trade-off, however, lies not in this general balance of the positive vs. negative facets of the instructor image, but in the relative balance of the cognitive engagement and distraction management on the part of the learner, that is, that the socio-emotional processing and cognitive engagement benefits must be sufficiently strong to outweigh the negative effect of distraction, to result in a clear effect of the instructor’s social presence for these learners. This would also help us account for the individual differences underlying the eye–brain–learning correspondence patterns we have discussed thus far.

In short, the instructor’s social presence entails both motivating and distracting facets, resulting from simultaneously heightened attentional control and socio-emotional processing demands. The trade-off hypothesis we propose here is focused on the substantial socio-emotional processing that is needed to stimulate more in-depth processing of the learning content relative to the attentional demands. When the cognitive and motivational benefits of the presence of an onscreen instructor outweigh the attentional costs of that presence (arising from secondary processing which distracts from the main learning task), then positive learning outcomes would emerge. Our study indicates that learners who exhibit more aligned brain–eye–learning correspondence can better capitalize on the benefit of the social presence of the human instructor and better manage visual distraction. By contrast, those who exhibit greater variability in brain–eye–learning synchronization are less able to fend off the distraction of the instructor’s image and therefore cannot fully leverage the benefit of the instructor’s onscreen presence.

For future studies of multimedia learning in this direction, we suggest that researchers incorporate other social cues that are commonly available in real-life teaching and learning situations, such as pointing, gesture, gaze direction changes, and movements (7). Future studies should also investigate how the difficulty or complexity of the learning content interacts with socio-emotional cues and differentially recruits neurocognitive resources. For example, more rudimentary or more familiar lecture materials may not as strongly engage visual attention, thus mediating the trade-off between attention and socio-emotional processing in relation to learning performance (9). In addition, neurocognitive studies across diverse learning environments, such as online platforms and virtual reality, could offer invaluable insights for optimizing multimedia learning experiences [see a recent study (55) testing various virtual platforms for remote collaboration in online learning].

To conclude, this study provides a systematic multimodal fMRI-eye tracking study for evaluating the key principles of multimedia learning. Our findings support a learning benefit in having onscreen presence of the instructor (even an animated instructor) for online video lectures whenever possible, given the convergent neurocognitive and behavioral evidence reported. Our findings hold significant implications for today’s education in a digital era, where online video learning is prevalent.

## Materials and Methods

### Participants.

Eighty-one college students (43 women and 39 men; mean age 24.8, range 21 to 34 y of age) from the Hong Kong Polytechnic University participated in this study. All participants were native speakers of Mandarin and reported having normal hearing and normal or corrected-to-normal vision. Participants had no history of mental or neurological disorders. The study was approved by the Institutional Review Board of The Hong Kong Polytechnic University. All participants gave written informed consent before taking part in the experiment. They received monetary compensation for their participation.

To ensure that learning could occur, we did not recruit any participants majoring in biology or meteorology due to the nature of the learning content (see details below). Data from eight participants were excluded from the statistical analyses reported below due to nausea, extreme sleepiness, or excessive head movement in the scanner. The final sample reported below included 73 participants (39 women and 34 men, mean age 24.6 y, range 19 to 31 y of age).

This study adopted a mixed design, with Topic as a within-subject factor and Group as a between-subject factor, to avoid familiarity effects and interference from different video formats and maximize the efficient use of data that could be gained from each participant. We recruited four groups of participants who took the video lectures that contained four different topics in the following conditions (Fig. 1*B*): instructor-absent condition (19 participants), instructor-present/neutral human condition (20 participants), instructor-present/smiling human condition (18 participants), and animated instructor condition (16 participants). To test the image principle, we contrasted the instructor-absent (19 participants) condition with the two instructor-present conditions (concatenating the instructor-present/neutral human and instructor-present/smiling human conditions; 38 participants). To test the embodiment principle, we contrasted the human instructor (18 participants) and animated instructor (16 participants) conditions; the former included the smiling human condition only to be comparable with the animated instructor that had only the smiling facial expression. A summary of the demographic information of the four groups of participants can be found in *SI Appendix*, Table S2.

### Materials.

Four texts covering the topics of lightning, memory, infectious disease, and the respiratory system served as the learning content of the study. These texts elaborate on the definition, classification, and formation of the themes (see variables controlled for these texts in *SI Appendix*, S4).

Sixteen videos were created for the four topics according to our experimental conditions: 1) lecture videos without an instructor (instructor-absent), with only the learning content (images and text) (Fig. 1*B*); 2) lecture videos with a human instructor with a neutral facial expression (instructor-present/neutral human), in which the instructor stood behind the podium to face the students when delivering the learning content (Fig. 1*B*); 3) lecture videos with a human instructor with a smiling facial expression (instructor-present/smiling human) while other aspects were the same as in condition 2) (Fig. 1*B*); 4) lecture videos with an animated instructor (instructor-present/animated) with a smiling facial expression, while other aspects of the condition were the same as in condition 3) (Fig. 1*B*). To ensure our participants received the learning content in the same way, we asked the same female speaker (the human instructor in condition 2) to record all four lecture audios (each audio file lasting approximately 150 s). The 16 videos consisted of presentation slides on the right and a podium on the left, in line with most multimedia learning experimental setups (3). All videos used a classroom-like setting as the background.

### Behavioral Measurements.

#### Prior knowledge test and post-test.

As in other multimedia learning studies (3), we tested participants’ prior knowledge to assess its potential impact on learning (*SI Appendix*, S5) and adopted three standard post-tests quantifying comprehension, recall, and transfer, respectively (see specific questions and scoring schemes in *SI Appendix*, S5).

#### Cognitive tests.

Our study tested individual learners’ attentional control, socio-emotional ability, and working memory (see details in *SI Appendix*, S5) as indicators of individual differences. We adopted the Attention Network Test (ANT) to assess attentional control (56), the Trait Emotional Intelligence Questionnaire-Short Form (TEI) to measure sociability and emotionality (57, 58), and the Letter Number Sequencing (LNS) test to measure working memory (59).

### Procedure.

The experiment included two separate visits to the laboratory. During their first visit, participants completed the prior knowledge test, followed by a practice session outside the MRI scanner. Once in the MRI scanner, they underwent a structural MRI scan before taking the main multimedia learning session, during which simultaneous fMRI and eye-tracking data were recorded using the fixation-related fMRI scanning paradigm (60, 61). This session included four separate functional runs, each consisting of watching a lecture video (150 s) followed by comprehension questions (true/false judgments). Participants were instructed to pay full attention to the lecture video and use an MRI-compatible button box to answer questions. The lecture videos and comprehension tests were presented using E-prime 3.0 (62), with the order of the four lecture videos randomized for each participant and counter-balanced across conditions. The in-scanner experiment lasted for about 40 min in total. After participants exited the scanner, they completed post-learning assessment including the recall and transfer tests. One week later, they were assessed with the battery of cognitive and socio-emotional tests described above. See Fig. 1*A* for illustration.

### Data Acquisition.

#### Eye-movement data.

Participants’ eye movements during in-scanner multimedia learning were simultaneously recorded with their neural responses via a long-range mount MRI-compatible eye tracker (Eye-Link 1000 Plus; SR-Research). The eye-tracker camera was positioned at the rear part of the scanner bore to record participants’ eye movements via a reflective mirror placed above the head coil. The distance between the camera and the participant’s eyes reflected on the reflective mirror was 120 cm. The recording was monocular based on the participants’ right eye movement. We performed a 13-point calibration and validation on the participants’ eyes, followed by a drift check. Drift checks repeated before video watching and question answering.

#### MRI data.

Data were acquired using a 3T Siemens Magnetom Prisma Fit scanner with a 64-channel head coil. After an initial localizing scan, an anatomical T1-weighted MPRAGE image was collected (176 ascending sagittal slices, FOV = 256 mm, TR = 1570 ms, TE = 2.15 ms, acquisition time = 220 s, flip angle = 9°, GRAPPA in-plane acceleration factor = 2). After the T1, four runs of functional images were acquired using a multiband T2* weighted echo-planar sequence (34 interleaved axial slices, TR = 1 s, TE = 30 ms, slice thickness = 3.5 mm, FOV= 240 × 240 mm^2^, and 80 × 80 matrix size with a resolution of 3 × 3 mm^2^, inter-slice gap = 10% of the slice thickness, flip angle = 58°, acquisition time = 182 s ~ 360 s (depending on self-paced question answering speed), multiband acceleration factor = 2, coverage omitted the lower extent of the cerebellum). Additionally, we acquired GRE field maps (short TE = 4.92 ms, long TE = 7.38 ms) for geometric distortion correction of EPI images.

### Data Analysis.

#### Behavioral data processing and analysis.

Learning performance was assessed using comprehension (in scanner), recall, and transfer scores (outside scanner; see *Procedure*). Two groups with two raters in each group scored the recall and transfer tests (*SI Appendix*, S5), with inter-rater reliabilities of *r* = 0.92 and *r* = 0.85, respectively.

#### Eye-tracking data processing and analysis.

The eye-tracking data were analyzed using the software Eyelink Data Viewer (Version: 3.2.1; SR Research Ltd). Two rectangular area of interests or AOIs (Fig. 1*B*) were constructed, which enclosed the instructor and the content slides (i.e., the learning content). Four fixation-based measurements were computed for each AOI: 1) the dwell time of each AOI, defined as the summed duration across all fixations that fell within the AOI; 2) dwell time percentage, defined as the percentage of dwell time in the AOI in relation to the summed duration of all fixations throughout the lecture video; 3) fixation count, defined as the total number of fixations falling within one AOI; and 4) fixation percentage, defined as the percentage of fixation counts for the AOI in relation to the summed fixation counts throughout the lecture video.

In order to examine the effect of the presence of the instructor on participants’ attention during learning, we conducted four ANCOVAs on the four fixation measurements of the instructor AOI, with covariates including age, gender, prior knowledge, undergraduate major, and postgraduate major. Similarly, to examine the effect of the embodiment type of the instructor, we performed eight ANCOVAs on the slide and instructor AOIs.

Following the approach of two recent studies (29, 30), we computed ISC of moment-to-moment eye movements under individual learning conditions using the leave-one-out method: 1) we extracted each participant’s moment-to-moment eye gaze positions in the vertical and horizontal coordinates during video watching, 2) we computed the Pearson correlation between the eye gaze position of each participant and the average eye gaze position of all other participants, respectively, for vertical and horizontal directions, 3) we averaged the two ISC values from the vertical and horizontal coordinates of eye gazes for each participant to derive an individual eye-movement ISC score for the following analysis. Before computing the eye-movement ISC, we utilized linear interpolation to make up for the missing data caused by eye blinks (30). Data from three participants were excluded from the ISC analysis due to incomplete eye movement data. Next, we performed two ANCOVAs with the same set of covariates to separately examine the effects of the onscreen presence of the instructor and the embodiment type of the instructor. We further correlated individual eye-movement ISCs with three learning performance measurements (comprehension, recall, and transfer) using Pearson correlation (one-tailed). Finally, we merged the different learning conditions to compute the correlation between individual eye-movement ISCs with the three learning performance measurements, to discern the extent to which eye-movement ISC could be predictive of learning outcomes across conditions.

#### fMRI data preprocessing and analysis.

The fMRI data preprocessing was conducted using the software SPM12 (Wellcome Centre for Human Neuroimaging; https://www.fil.ion.ucl.ac.uk/spm/software/spm12/). We adopted the following preprocessing pipelines: 1) discarding the first 11 volumes (17); 2) performing slice timing corrections; 3) creating voxel displacement maps followed by realignment and unwarping; 4) conducting coregistration of the anatomical data to the mean functional image; 5) normalizing all functional images to MNI space; 6) spatially smoothing the functional images by applying a 6 mm full-width-at-half-maximum Gaussian kernel. Head motion was quantified by three translational and three rotational parameters. One participant’s data were excluded from further analysis based on the exclusion criteria (>3 mm translation or >3° rotation).

Smoothed functional images were entered into a first-level analysis to regress out six motion parameters, and a 128 s high-pass filter was applied to remove low-frequency signals. The resulting residuals of all functional images from four separate runs were concatenated into a single image in the same order across all subjects for the following analyses.

#### ISC.

We assessed shared neural responses among learners under each learning condition using the ISC analysis (16–18) (Fig. 1*C*). The leave-one-out method was used to compute ISC at the subject level within each condition: Each participant’s time-series data were correlated with the average of the remaining participants’ time-series data, yielding one ISC map for each subject. Individual ISCs were computed for each voxel within the group-level gray-matter mask. We performed Fisher’s z-transformation to normalize the voxel-wise correlation coefficients for the following analyses.

After obtaining individual learners’ ISCs, we derived group-level ISC maps for each condition. In order to obtain the group-level ISC map, we conducted four one-sample *t* tests using SPM with covariates including learners’ age, gender, prior knowledge, undergraduate major, and postgraduate major. For the statistical thresholding of the SPM analyses, we set the maximum statistical threshold as puncorr <0.001 at the voxel level and then took a cluster size correction threshold pcluster of qFDR < 0.05 determined at puncorr <0.001. Finally, we reported only the clusters with at least ten voxels. Further at the group level, we performed two sets of two-samples *t* tests. The statistical thresholds were also kept consistent. We used BrainNet Viewer (63) to visualize the results.

#### Whole-brain IS-RSA for cognitive and socio-emotional processing.

The whole-brain IS-RSA is operationalized by calculating Spearman’s rank-ordered correlations between the vectorized off-diagonal triangles of two subject-by-subject similarity matrices (26, 28, 31). These two similarity matrices were respectively constructed based on pairwise brain ISC matrix and pairwise behavioral similarity matrices derived from cognitive tests (i.e., accuracy score from conflict monitoring of the ANT and total score from LNS) and sociability and emotionality scores from Chinese TEI (*Behavioral Measurements*). For the behavioral similarity matrices grounded on these abilities, the computation of pairwise similarity between any two pairs of subjects allowed higher scores to generate higher similarity compared with subjects with lower scores (28; see *SI Appendix*, S6 for details). Since matrix constructed upon sociability scores did not correlate with brain ISC matrices in any brain regions, we discarded it from further analysis and focused on emotionality scores.

The statistical significance for the brain–behavior representational similarity was determined by the non-parametrical Mantel test (64). When conducting the Mantel test, we randomly permutated subject labels of the brain ISC (i.e., rows and columns of the brain ISC matrix) 10,000 times to obtain a null distribution of surrogate Spearman’s correlation coefficients. Then we compared our observed Spearman’s correlation coefficient to this null distribution to obtain a *P*-value for each voxel. These *P*-values were then FDR corrected due to considerations of multiple comparison. For those voxels that survived the FDR correction, we further set a cluster-size threshold of 10 survived voxels and extracted MNI coordinates of the first and second peaks to create ROIs with a radius of 5 mm for our subsequent ROI-based IS-RSAs.

#### ROI-based IS-RSA for learning performance.

To identify brain–learning outcome correlations, we further performed a series of ROI-based IS-RSA of our learning performance data comprised of recall, comprehension, and transfer scores with the key ROIs from the whole-brain voxel-wise IS-RSA. When constructing behavioral matrices for the learning performance data, we were open to entertain two possible scenarios when constructing behavioral matrices, that is, neural responses may be similar and clustered together for participants a) either at the higher end of a certain behavioral spectrum or b) at a similar position anywhere along a behavioral spectrum (28). In total, we created six behavioral matrices embedding these two pairwise similarity measurements (see *SI Appendix*, S6 for details) with learners’ recall, comprehension, and transfer scores.

For ROI-based brain matrices, we extracted the neural responses of ROIs by averaging neural signals across all voxels. We then constructed a pairwise brain ISC matrix for each ROI among learners under each condition. Next, we calculated Spearman’s correlation coefficients between the ISC matrix of each ROI and six learning performance matrices. Finally, as with the whole-brain IS-RSA, we performed Mantel tests with 10,000 permutations to determine the significance of these correlations.

We conducted an additional analysis to determine whether higher-achieving learners allocated more eye fixations to the human instructor or to the animated instructor compared to lower-achieving learners. To do this, we derived an overall learning performance score by concatenating learners’ recall, comprehension, and transfer scores. Learners were then divided into higher- and lower-achieving groups based on the median of the overall learning performance score. Two-sample *t* tests were applied to compare the fixation-based measurements of attention to the human or the animated instructor between the higher vs. lower-achieving learners.

#### Correlational analysis for eye-movement ISC and brain ISC.

In order to bridge our multimedia learning outcome with our multimodal data encompassing eye movements and neural activities, we computed Pearson correlation (one-tailed) between eye-movement ISC scores with brain ISC scores. We used the leave-one-out approach to compute the eye-movement ISC and brain ISC scores, so that each single ISC indicates the synchronization of eye movements or that of neural activities between this participant with the rest of the group. In this analysis, we only focused on brain regions implicated in cognitive/socio-emotional processing aspects of multimedia learning where neural synchrony correlated with learning performance matrices. In addition, we computed Spearman correlations between pairwise brain ISC matrices of the aforementioned regions with the eye-movement ISC matrices using the IS-RSA approach. To determine the significance of the correlation between the two matrices, we performed Mantel tests with 10,000 permutations (64). FDR correction (q < 0.05) was applied to all the correlation analyses to control for type I error in our multiple comparisons.

## Supplementary Material

Appendix 01 (PDF)

## Data Availability

Anonymized behavioral, eye-tracking, and fMRI data have been deposited in the Open Science Framework (OSF) data repository (https://osf.io/tc5bq/) (65). The program codes for the IS-RSAs reported in the *Materials and Methods* section are also available on this OSF website (https://osf.io/tc5bq/).

## Footnotes

^*^The human instructor condition here included data from only the instructor-present/smiling human sub-group, so that the results could be compared with those from the animated instructor condition (which included only smiling animated instructors).
