# Supplementary material for: Onscreen presence of instructors in video lectures affects learners’ neural synchrony and visual attention during multimedia learning
Source: Proc Natl Acad Sci U S A. 2024 Mar 11;121(12):e2309054121. doi: 10.1073/pnas.2309054121 (PMC10963011; doi:10.1073/pnas.2309054121)
Supplement: Supplementary file 1 — Appendix 01 (PDF) [file pnas.2309054121.sapp.pdf]

PNAS

Supporting Information for

Onscreen Presence of Instructors in Video Lectures Affects Learners' Neural Synchrony and Visual Attention During Multimedia Learning

Chanyuan Gu[a1], Yingying Peng[a1], Samuel A. Nastase[b], Richard E. Mayer[c], Ping Li[a,d*]

[a] Department of Chinese and Bilingual Studies, Faculty of Humanities, The Hong Kong Polytechnic University; [b] Princeton Neuroscience Institute and Department of Psychology, Princeton University; [c] Department of Psychological and Brain Sciences, University of California, Santa Barbara; [d] Centre for Immersive Learning and Metaverse in Education, The Hong Kong Polytechnic University

* Ping Li
Email: pi2li@polyu.edu.hk

This PDF file includes:

Tables S1 to S8
Supporting texts S1 to S6
Figure S1 to S2
References

[1] C.G. and Y.P. contributed equally to this work

Table S1. Abbreviations for Brain Regions

Abbreviation	Brain Region
ACC	Anterior cingulate cortex
AG	Angular gyrus
aMCC	Anterior midcingulate cortex
DLPFC	Dorsolateral prefrontal cortex
dmPFC	Dorsal medial prefrontal cortex
dPPC	Dorsal posterior parietal cortex
FG	Fusiform gyrus
FEF	Frontal eye fields
IFG	Inferior frontal gyrus
IPL	Inferior parietal lobe
IPS	Inferior parietal sulcus
LG	Lingual gyrus
MCC	Middle cingulate cortex
MFG	Middle frontal gyrus
MOG	Middle occipital gyrus
MTG	Middle temporal gyrus
PCG	Posterior cingulate gyrus
PFC	Prefrontal cortex
PPC	Posterior parietal cortex
SFG	Superior frontal gyrus
SPL	superior parietal lobule
STG	Superior temporal gyrus
STS	Superior temporal sulcus
TP	Temporal pole
TPJ	Temporoparietal junction

Table S2. Demographic information of all participants under the learning conditions (M ± SD)

	Instructor-absent (19)	Instructor-present/ neutral human (20)	Instructor-present/ smiling human (18)	Smiling animated instructor (16)
Age	24.8 (3.25)	24.8 (3.47)	25 (3.33)	23.4 (3.27)
Gender	9 Female<br>10 Male	11 Female<br>9 Male	11 Female<br>7 Male	7 Female<br>9 Male
Prior knowledge	11.2 (3.27)	12.6 (4.14)	11.3 (2.96)	13.6 (5.18)

Note: M and SD (in parentheses) indicate the average and standard deviations respectively.

Table S3. Examples of prior knowledge, comprehension, and transfer questions

Tests	Examples
Prior knowledge	I often read weather forecasts reports.
Comprehension	Electric charge is formed by friction and collision between updraft and downdraft.
Transfer	Please write down at least three factors that impact the intensity of lightning based on the formation processes of lightning.

Table S4. The ANCOVA results of Instructor type for learning performance

Learning performance	Instructor type				*F* test	Post-hoc test
	Instructor-absent (A)	Instructor-present/ neutral human (B)	Instructor-present/ Smiling human (C)	Smiling animated instructor (D)		
Comprehension	.73<br>(0.08)	.81<br>(0.04)	.81<br>(0.05)	.80<br>(0.11)	4.20**	A < B*<br>A < C*<br>B = C = D
Recall	4.16<br>(1.09)	4.90<br>(0.93)	5.10<br>(1.10)	5.32<br>(1.23)	2.58	
Transfer	.54<br>(0.10)	.62<br>(0.09)	.64<br>(0.10)	.70<br>(0.08)	5.49**	A < B*<br>A < C*<br>A < D**<br>B = C = D

Note: The post-hoc model estimation by the 'emmeans' R package failed when categorical variables (e.g., major and gender) were added to the model, so we only included continuous variables including age and prior knowledge as covariates; M and SD (in parentheses) indicate the average and standard deviations respectively. $^{**}p < .01$; $^{*}p < .05$.

Table S5. Group comparisons of ISCs for the image principle and embodiment principle, respectively. Table corresponds to Fig. 2C and Fig. 2F

H	Region	BA	Voxels (K)	MNI x	y	z	*T*
Instructor-present > Instructor-absent							
L	Fusiform Gyrus	21	11	-54	-10	-28	4.32
R	Medial Prefrontal Cortex/Anterior Cingulate	32/10	100	42	38	-10	6.09
R	Superior Temporal Gyrus	42	29	60	-28	14	5.85
R	Middle Temporal Gyrus	22	12	42	-4	-16	4.42
R	Superior Frontal Gyrus	8	13	18	26	56	4.49
Animated instructor > Human instructor							
R	Fusiform Gyrus	19	25	30	-64	-16	5.89
R	Middle Occipital Gyrus	19	28	42	-82	14	5.48
L	Lingual Gyrus	18	21	0	-76	-4	5.72
L	Middle Temporal Gyrus	37	28	-54	-52	-4	6.79
L	Middle Frontal Gyrus	45/56	12	-48	20	26	5.39
L	Superior Parietal Lobule	7	23	-30	-70	44	4.99
R	Inferior Frontal Gyrus	46	17	48	32	14	5.21

Note: The coordinates in MNI space of the peak activations in clusters and corresponding T values were listed ($q_{FDR} < .05$, cluster-level correction). H = hemisphere; L = left hemisphere; R = right hemisphere; BA = Brodmann areas.

S1. Comparable neural synchronization between the neutral and smiling human instructor conditions

To assess neural synchronization across learners under the neutral human instructor and smiling human instructor learning conditions, we constructed two group-level ISC maps using the leave-one-out intersubject correlation (ISC) approach. We identified significant ISCs in visual and auditory regions, high-order language regions, and frontoparietal executive control regions under both learning conditions (see Figure S1). We conducted a two-sample t-test (controlling for the same set of covariates as other group t-tests) to compare the ISCs between the two learning conditions. The results indicated that learners under the neutral and smiling human instructor learning conditions exhibited comparable neural synchronization across the whole brain. Combined with the comparable learning performance under the two conditions (see Table S4), these findings jointly revealed the null effect of the facial expression of instructor on the learner's behavior and neural activities during learning. Hence, we concatenated the learners from the neural and smiling human instructor learning conditions to form the instructor-present group for the main analyses.

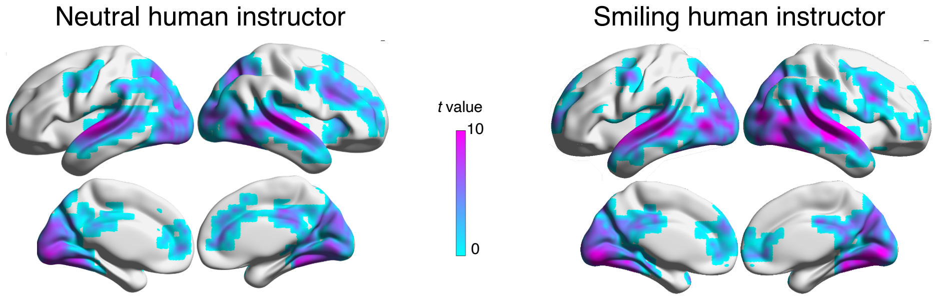

Figure S1. Brain regions with significant neural synchronizations among learners under the neutral human instructor (left) and smiling human instructor (right) conditions

S2. Hierarchical regression models to evaluate additional predictive power of either brain ISC or eye-movement ISC on learning performance

We conducted a supplementary hierarchical regression analysis on the eye-movement ISC and brain ISC patterns to assess if brain or eye-movement ISC would have additional predictive power over and above one another on learning performance. These analyses are intended to help us better elucidate the three-way relationships among the brain ISC, the eye-movement ISC, and the learning performance.

Specifically, we focused on the brain ISC in brain regions implicated in socio-emotional and working memory processing (i.e., left cuneus, right precuneus, and right LG), which have been shown to be associated with both the eye-movement ISC and participants' learning performance under the instructor-present condition (Results section, final part). Given the nature of hierarchical regression analysis, we constructed two types of models: one model in which eye-movement ISC was included in the first step, and then brain ISC added in the second step; and another model in which brain ISC was included in the first step, and then eye-movement ISC added in the second step. To simplify our modelling task, we selected the comprehension scores to be our dependent variable in both types of models.

Overall, the model including only eye-movement ISC was significant ($F_{1,35}$ = 4.29, p = .046, R^2 = .11; see Table S6). Further, the model that included the eye-movement ISC plus the brain ISC in the right precuneus was significant ($F_{2,34}$ = 3.40, p = .045, R^2 = .17). However, the increased fit of the second model over the first model did not reach statistical significance (ΔF = 2.35, p = .13, ΔR^2 = .06; see Table S6). Interestingly, we saw a similar pattern when the regression analysis started with the brain ISC and then included the eye-movement ISC. Specifically, the first model with only the brain ISC in the right precuneus was significant ($F_{1,35}$ = 4.64, p = .038, R^2 = .12). The second model with both the brain ISC and the eye-movement ISC was also significant ($F_{2,34}$ = 3.40, p = .045, R^2 = .17), but the improvement from the first model to the second model was not significant (ΔF = 2.03, p = .16,

$\Delta R^2 = .05$; see Table S6). In both cases, we saw an increase in a model's ability to account for more variance when the model included both types of ISC, as compared with the model that had only one or the other information. Although the increase in the additional variance accounted for by either brain ISC or eye-movement ISC alone did not reach statistical significance in the present study, this could be due to the high collinearity between the two types of ISC data; hierarchical regression models are notoriously sensitive to collinearity (1).

Given the above analyses, we suggest that the brain ISC is not merely a 'read out' of where the learners look, and at the same time, the brain ISC also does not capture every pattern in the eye-movement ISC, either. These analyses prompted us to consider treating brain synchronization and eye-movement synchronization as partially overlapping, rather than causally linked, indices in the context of multimedia learning. At a more general level, brain and eye-movement data may both capture some unique information that contributes to learning, and both are correlated with one another and with students' learning performance. Consistent with previous literature (2), we suggest that our participants displayed the alignment of eye-movement ISC and brain response ISC that reflects the synchronization of multiple signals and their complex dynamics modulated by the effective cognitive processing of the video lecture stimuli.

Table S6. Hierarchical model summary for eye-movement ISC (first step) and brain ISC in right precuneus (second step)

Model	Predictor	R^2	F	ΔR^2	Beta
First model		.11	4.29*	NA	
	Eye-movement ISC				.22*
Second model		.17	3.40*	.06	
	Eye-movement ISC				.16
	Brain ISC				.16

Note: *p < .05.

Table S7. Hierarchical model summary for brain ISC in right precuneus (first step) and eye-movement ISC (second step)

Model	Predictor	R^2	F	ΔR^2	Beta
First model		.12	4.64*	NA	
	Brain ISC				.22*
Second model		.17	3.40*	.05	
	Brain ISC				.16
	Eye-movement ISC				.16

Note: *p < .05.

S3. Greater neural synchronization under the animated instructor condition compared to the instructor-absent condition

To better understand the effect of instructor's social presence in the form of an animated instructor on learners' neural responses, we additionally compared the group-level ISC maps between the animated instructor and the instructor-absent learning conditions via a two-sample t-test (controlling for the same set of covariates as other group t-tests). We identified greater neural synchronization in the right lingual gyrus, right superior temporal gyrus, left middle temporal gyrus, and left precuneus under the animated instructor learning condition compared with the instructor-absent learning condition (see Figure S2 and Table S8), but not in the FG area, a classical face processing brain region (3). In contrast, we did not observe any brain region showing greater neural synchronization under the instructor-absent learning condition.

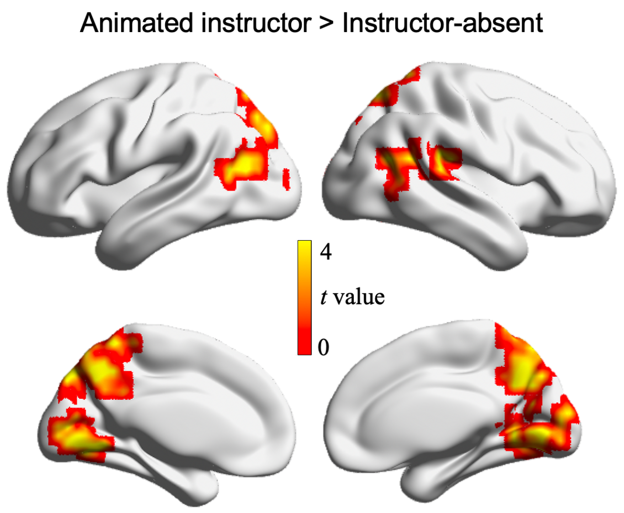

Figure S2. Brain regions with greater neural synchronizations among learners under the animated instructor (left) condition compared with the instructor-absent condition.

Table S8. Group comparisons of ISCs under the animated instructor vs. instructor-absent learning conditions

H	Region	BA	Voxels (K)	MNI x	y	z	*T*
Animated instructor > Instructor-absent							
R	Lingual gyrus	18	100	6	-58	2	7.24
R	Superior Temporal Gyrus	39	28	48	-64	20	4.85
L	Middle Temporal Gyrus	13/39	18	-48	-70	14	5.20
L	Precuneus	7	108	-6	-58	44	5.69

Note: The coordinates in MNI space of the peak activations in clusters and corresponding T values are shown (qFDR < .05, cluster-level correction). H = hemisphere; L = left hemisphere; R = right hemisphere; BA = Brodmann areas.

S4. Variables controlled for the four video scripts

Following practices in psycholinguistics and reading comprehension, we controlled for the following variables for the scripts of the four videos: the length of texts (mean = 508.25), number of characters (mean = 465.25 characters), mean number of strokes (mean = 7.36), mean length of sentences (mean = 19.18 characters) and the ratio of high-frequency words (mean = 0.72) across these four texts. These aspects were considered to reflect Chinese text difficulties (4).

S5. Behavioral Measurements

Prior knowledge tests. Participants rated on a 7-point Likert scale regarding the extent to which the descriptions matched their knowledge status for each topic, with 1 being '*not matching at all' and* 7 being '*matching very well'*. An example can be found in *SI Appendix*, Table S3. The mean value of summed ratings for each topic was considered to demonstrate participants' prior knowledge of each text/video of the lecture.

Post-tests. Following the traditional multimedia learning studies (5), we adopted three standard post-tests quantifying comprehension, recall, and transfer, which target participants' understanding, memorization, and their ability to utilize the acquired knowledge to resolve new problems, respectively. The comprehension test included four sets of fourteen true-false questions following the target learning video presentations. Participants were required to answer each question within 10 seconds based on the comprehension of the material (see *SI Appendix*, Table S3 for an example question). The average accuracy (ranging from 0 to 1) across the four sets of comprehension questions was treated as the comprehension score. Second, the recall test required participants to write down as much as they could remember from the lecture video within 3 minutes. For each text, there was a set number of pre-selected idea units or keywords such as "anions gradually decline with the downdraft" and "downdraft". For scoring, one point was given to one idea unit or one key word, and the average score reflected the accuracy for that target learning topic. Finally, the transfer test included four questions designed for each topic. These questions aimed to test participants' knowledge transfer after learning (see *SI Appendix*, Table S3 for an example question). Participants were given two minutes to complete each transfer question. Participants received one point if they answered a transfer question accurately, and the average accuracy (ranging from 0 to 1) obtained from all four topics was used as the transfer score.

Cognitive and socio-emotional tests

Attention Network Test (ANT). The ANT test (6) measures attentional networks including alerting, orienting, and conflict. It combines attentional and spatial cues with a flanker test. For each trial, a central arrow was presented with congruent or incongruent flanking arrows accompanied by attentional and/or spatial cues. The attentional cues were either double asterisks appearing on the upper and lower screen or single asterisk appearing in the center of the screen in some trials before the arrows. They served to alert participants that the arrows would appear soon. The spatial cues were asterisks either appearing on the upper or lower screen, to alert participants that the arrows would appear soon and orient participants' attention to the location of the arrows. Participants were required to indicate the direction of central arrows as fast and

accurately as possible. The accuracy and reaction time (RT) of the correct trials were used as the measurements. As indicated in Fan et al. (6), the altering was defined as the difference between double- cue and no-cue trials in accuracy and RT; the orienting effect was defined as the difference between spatial- cue and central-cue trials; the conflict effect was defined as the difference between congruent and incongruent trials. Taking the conflict effect for instance, smaller difference in accuracy denotes, better conflict monitoring ability.

Letter Number Sequencing (LNS). The LNS test adopted from the Wechsler Adult Intelligence Scale assesses short-term working memory (7). In this task, participants were auditorily presented with a series of randomly mixed combinations of numbers and letters. They needed to recall the numbers in ascending order followed by letters in alphabetical order immediately after each audio presentation by typing down their answers. Their answers were manually checked for misused keyboards such as extra blank between numbers and letters. The test began with two characters (one letter and one number) and was increased by one character every three trials, and the longest trials included eight characters. In total, there were 21 trials. One point was given for one correct response. The number of correct trials was used as the total score.

Trait Emotional Intelligence Questionnaire-Short Form (TEI). The Chinese-version TEI test (8) adopted from TEI-Que (9) measures trait emotional intelligence (TEI) and consists of 30 items grouped into four main factors: well-being, emotionality, sociability, and self-control. Participants were required to rate each item on a 7-point Likert scale from 1 (Completely Disagree) to 7 (Completely Agree). In this study, we only used the emotionality and sociability factor, because well-being and self-control may not directly influence the student's socio-emotional processing during learning. High scores on emotionality and sociability indicate a wide range of emotion-related skills and better ability in social interaction, respectively, whereas low scores indicate difficulties in perceiving and expressing emotional states and affecting others' emotions.

S6. Whole-brain and ROI-based IS-RSA models

In the whole-brain IS-RSA analysis linking brain data with cognitive/socio-emotional abilities, we adopted the 'Anna Karenina' or AnnaK principle (10). Specifically, we constructed pairwise similarity matrices where higher pairwise similarity emerges among subjects with higher behavioral ability scores. To do so, an absolute position on the behavioral scale for each subject was estimated by sorting behavioral measurements reflecting cognitive and socio-emotional processing abilities. The similarity (i.e., $S_{i,j}$) of each pair of subjects was computed using Equation (1), where P_i and P_j were the absolute positions of subjects *i* and *j* respectively and *N* was the number of subjects. Hence, $S_{i,j}$ calculated from the pairs of subjects with higher behavioral scores would be greater compared to $S_{i,j}$ obtained from the remaining pairs of subjects.

$$S_{i,j} = \frac{(P_i + P_j)}{2 * N} \qquad \text{Equation (1)}$$

In the ROI-based IS-RSA analysis linking brain data with learning performance data, we constructed two types of pairwise similarity matrices. The first type of similarity matrices followed the 'AnnaK' principle and was operationalized in the same way as the whole-brain IS-RSA analysis. As we did not hold any specific hypothesis regarding the brain response and learning performance, we also organized the pairwise similarity matrices in a way that higher pairwise similarity emerges from similar behavioral scores, which is named as the 'nearest-neighbor' or NN principle (10). We first obtained the absolute position on individual behavioral scale by sorting the learning outcomes measured by recall, comprehension, and transfer tasks. The similarity (i.e., $Sn_{i,j}$) of each pair of subjects was computed using Equation (2), where P_i and P_j indicated the absolute positions of subjects *i* and *j* respectively and *N* was the number of subjects. Therefore, $Sn_{i,j}$ estimated from the pairs of subjects with similar learning outcomes would be greater than $Sn_{i,j}$ calculated from the pairs of subjects with less similar learning outcomes.

$$Sn_{i,j} = 1 - \left|\frac{(P_i - P_j)}{N}\right| \qquad \text{Equation (2)}$$

SI References

1. N. Shrestha, Detecting multicollinearity in regression analysis. *Am. J. Appl. Math. Stat.* **8**, 39–42 (2020).
2. J. Madsen, L. C. Parra, Cognitive processing of a common stimulus synchronizes brains, hearts, and eyes. *PNAS Nexus* **1**, pgac020 (2022).
3. N. Kanwisher, J. McDermott, M. M. Chun, The fusiform face area: a module in human extrastriate cortex specialized for face perception. *J. Neurosci.* **17**, 4302–4311 (1997).
4. N. Zhao, X. Han, Study on text readability formula in Chinese reading assessment. *Exam. Res.* **4**, 23–29 (2017).
5. R. E. Mayer, *Multimedia Learning*, 3rd Ed. (Cambridge University Press, 2020) https:/doi.org/10.1017/9781316941355.
6. J. Fan, B. D. McCandliss, T. Sommer, A. Raz, M. I. Posner, Testing the efficiency and independence of attentional networks. *J. Cogn. Neurosci.* **14**, 340–347 (2002).
7. D. Wechsler, *WMS-III Administration and Scoring Manual* (TX: The Psychological Corporation, 1997).
8. A. Feher, G. Yan, D. H. Saklofske, R. A. Plouffe, Y. Gao, An Investigation of the Psychometric Properties of the Chinese Trait Emotional Intelligence Questionnaire Short Form (Chinese TEIQue-SF). *Front. Psychol.* **10** (2019).
9. K. V Petrides, "Psychometric properties of the trait emotional intelligence questionnaire (TEIQue)" in *Assessing Emotional Intelligence*, J. D. A. Parker, D. H. Saklofske, C. Stough, Eds. (Springer, 2009), pp. 85–101.
10. E. S. Finn, *et al.*, Idiosynchrony: From shared responses to individual differences during naturalistic neuroimaging. *Neuroimage* **215**, 116828 (2020).
